# Genome-Wide Transcription Factor Binding in Leaves from C_3_ and C_4_ Grasses

**DOI:** 10.1105/tpc.19.00078

**Published:** 2019-08-19

**Authors:** Steven J. Burgess, Ivan Reyna-Llorens, Sean R. Stevenson, Pallavi Singh, Katja Jaeger, Julian M. Hibberd

**Affiliations:** a Department of Plant Sciences, University of Cambridge, Cambridge CB2 3EA, United Kingdom; b Sainsbury Laboratory, University of Cambridge, Cambridge CB2 1LR, United Kingdom

## Abstract

The majority of plants use C_3_ photosynthesis, but over 60 independent lineages of angiosperms have evolved the C_4_ pathway. In most C_4_ species, photosynthesis gene expression is compartmented between mesophyll and bundle-sheath cells. We performed DNaseI sequencing to identify genome-wide profiles of transcription factor binding in leaves of the C_4_ grasses *Zea mays*, *Sorghum bicolor*, and *Setaria italica* as well as C_3_ *Brachypodium distachyon*. In C_4_ species, while bundle-sheath strands and whole leaves shared similarity in the broad regions of DNA accessible to transcription factors, the short sequences bound varied. Transcription factor binding was prevalent in gene bodies as well as promoters, and many of these sites could represent duons that influence gene regulation in addition to amino acid sequence. Although globally there was little correlation between any individual DNaseI footprint and cell-specific gene expression, within individual species transcription factor binding to the same motifs in multiple genes provided evidence for shared mechanisms governing C_4_ photosynthesis gene expression. Furthermore, interspecific comparisons identified a small number of highly conserved transcription factor binding sites associated with leaves from species that diverged around 60 million years ago. These data therefore provide insight into the architecture associated with C_4_ photosynthesis gene expression in particular and characteristics of transcription factor binding in cereal crops in general.

## Abstract (precis)

Genome-wide patterns of transcription factor binding in vivo can be defined using DNaseI in leaves of C_3_ and C_4_ grasses.

## INTRODUCTION

Most photosynthetic organisms, including crops of global importance such as wheat (*Triticum aestivum*), rice (*Oryza sativa*), and potato (*Solanum tuberosum*), use the C_3_ photosynthesis pathway in which Ribulose Bisphosphate Carboxylase Oxygenase (Rubisco) catalyzes the primary fixation of CO_2_. However, carboxylation by Rubisco is competitively inhibited by oxygen binding the active site (Bowes et al., 1971). This oxygenation reaction generates toxic waste products that are recycled by an energy-demanding series of metabolic reactions known as photorespiration (Tolbert, 1971; Bauwe et al., 2010). The ratio of oxygenation to carboxylation increases with temperature (Jordan and Ogren, 1984; Sharwood et al., 2016), and so losses from photorespiration are particularly high in the tropics.

Multiple plant lineages have evolved mechanisms that suppress oxygenation by concentrating CO_2_ around Rubisco. One such strategy is known as C_4_ photosynthesis. Species that use the C_4_ pathway include maize (*Zea mays*), sorghum (*Sorghum bicolor*), and sugarcane (*Saccharum officinarum*), and they represent the most productive crops on the planet (Sage and Zhu, 2011). In C_4_ leaves, additional expenditure of ATP, alterations to leaf anatomy and cellular ultrastructure, and spatial separation of photosynthesis between compartments (Hatch, 1987) allow CO_2_ concentration to be increased around 10-fold compared with that in the atmosphere (Furbank, 2011). Despite the complexity of C_4_ photosynthesis, it is found in over 60 independent plant lineages (Sage et al., 2011). In most C_4_ plants, the initial Rubisco-independent fixation of CO_2_ and the subsequent Rubisco-dependent reactions take place in distinct cell types known as mesophyll and bundle-sheath cells. Although the spatial patterning of gene expression that generates these metabolic specializations is fundamental to C_4_ photosynthesis, very few examples of *cis*-elements or *trans*-factors that restrict gene expression to mesophyll or bundle-sheath cells of C_4_ plants have been identified (Gowik et al., 2004; Brown et al., 2011; Williams et al., 2016; Reyna-Llorens et al., 2018). Moreover, in grasses more generally, the DNA binding properties of relatively few transcription factors have been validated (Bolduc and Hake, 2009; Eveland et al., 2014; Pautler et al., 2015; Yu et al., 2015). In summary, in both C_3_ and C_4_ species, work has focused on analysis of mechanisms controlling the expression of individual genes, and so our understanding of the overall landscape associated with photosynthesis gene expression is poor.

In yeast and animal systems, the high sensitivity of open chromatin to DNaseI (Zentner and Henikoff, 2014) has allowed comprehensive, genome-wide characterization of transcription factor binding sites at single-nucleotide resolution (Hesselberth et al., 2009; Neph et al., 2012; Thurman et al., 2012). In plants, DNaseI sequencing (DNaseI-seq) and more recently assay for transposase-accessible chromatin have been employed in C_3_ species and provided insight into the patterns of transcription factor binding associated with development (Zhang et al., 2012a, 2012b, 2016; Pajoro et al., 2014), heat stress (Sullivan et al., 2014), and root cell differentiation (Maher et al., 2018). By carrying out DNaseI-seq on grass leaves that use either C_3_ or C_4_ photosynthesis, we aimed to provide insight into the transcription factor binding repertoire associated with each form of photosynthesis. Our data indicate that more transcription factor binding sites are found in gene bodies than promoters, and up to 25% of the footprints represent “duons,” sequences located in exons that have an influence on both gene regulation and the amino acid sequence of the protein they encode. It is also clear that specific cell types from leaf tissue make use of a markedly distinct *cis*-regulatory code and that, despite significant turnover in the cistrome of grasses, a small number of transcription factor motifs are conserved across 60 million years of evolution. Comparison of sites bound by transcription factors in both C_3_ and C_4_ leaves demonstrates that the repeated evolution of C_4_ photosynthesis is built on both the de novo gain of *cis*-elements and the exaptation of highly conserved regulatory elements found in the ancestral C_3_ system.

**Figure:**
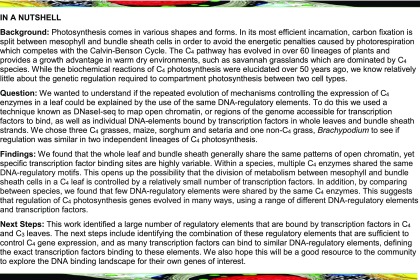


## RESULTS

### A *cis*-Regulatory Atlas for Grasses

**Figure 1.**
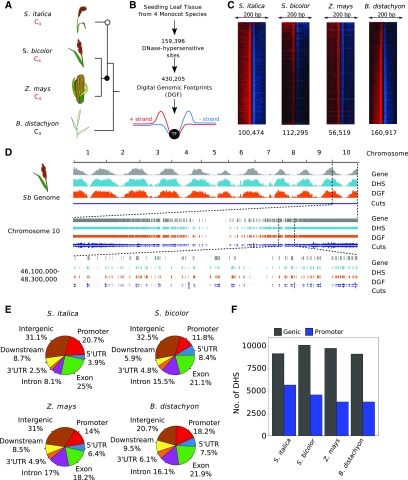
Transcription Factor Binding Atlas for Whole-Leaf Samples of Four Grasses. **(A)** Schematic of the phylogenetic relationship between the species analyzed. The two independent origins of C_4_ photosynthesis are highlighted with black and white circles (figure not drawn to scale). **(B)** Summary of sampling and the total number of DHSs and DGFs identified across all four species. **(C)** TreeView diagrams illustrating cut density around individual DGFs. Each row represents an individual DGF; cuts are colored according to whether they align to the positive (red) or negative (blue) strand and indicate increased cutting in a 50-bp window on either side of the DGF. The total number of DGFs per sample is shown at the bottom. **(D)** Representation of DNaseI-seq data from *S. bicolor*, depicting gene (gray), DHS (light blue), DGF (orange), and DNaseI (dark blue) cut density at three scales: genome-wide, with chromosome number and position indicated (top); chromosomal (second level); and kilobase genomic region (third level). Between the levels, the expanded area is denoted by dashed lines. **(E)** Pie charts representing the distribution of DHSs among genomic features. Promoters are defined as sequence up to 2000 bp upstream of the transcriptional start site, downstream represents regions 1000 bp downstream of the transcription termination site, while intergenic represents >1000 bp downstream of the transcription termination site until the next promoter region. UTR, untranslated region. **(F)** Bar chart representing the number of DHSs in genic and promoter regions.

To provide insight into the regulatory architecture associated with C_3_ and C_4_ photosynthesis in cereal crops, four grass were selected. *Brachypodium distachyon* uses the ancestral C_3_ pathway (Figure 1A). *S. bicolor*, *Z. mays*, and *Setaria italica* all use C_4_ photosynthesis; they were chosen because phylogenetic reconstructions indicate that *S. italica* represents an independent evolutionary origin of the C_4_ pathway (Figure 1A) and comparison of these species can provide insight into parallel and convergent evolution of C_4_ gene expression. Nuclei from a minimum of duplicate samples of *S. italica* (C_4_), *S. bicolor* (C_4_), *Z. mays* (C_4_), and *B. distachyon* (C_3_) leaves were treated with DNaseI (Supplemental Figure 1) and subjected to deep sequencing. A total of 806,663,951 reads could be uniquely mapped to the respective genome sequences of these species (Supplemental Data Set 1). From all four genomes, 159,396 DNaseI-hypersensitive sites (DHSs) of between 150 and 15,060 bp representing broad regulatory regions accessible to transcription factor binding were identified (Figure 1B). Between 20,817 and 27,746 genes were annotated as containing at least one DHS (Supplemental Data Set 2). For subsequent analysis, only DHSs that were consistent between replicates as determined by the irreproducible discovery rate framework (Li and Dewey, 2011) were used.

DNaseI footprinting is a well-established technique for detecting DNA-protein interactions at base-pair resolution and as such has been used to generate digital genomic footprints (DGFs) to predict transcription factor binding sites. DGFs are obtained by pooling all replicates to maximize the number of reads that map within each DHS and then modeling the differential accumulation of reads mapping to positive or negative strands around transcription factor binding sites within the DHSs (Piper et al., 2013). However, the DNaseI enzyme possesses some sequence bias that can affect the prediction of transcription factor binding sites (He et al., 2014; Yardımcı et al., 2014). After performing DNaseI-seq on “naked DNA” that is devoid of nucleosomes from each species, we identified hundreds of DGFs that likely represent false positives (Supplemental Figure 2A). For all species, analysis of the DGFs derived from naked DNA showed that treatment with DNaseI led to similar sequences being preferentially digested (Supplemental Figure 2B). However, because false-positive DGFs predicted from this approach will be influenced by the number of reads that map to each genome, and in the case of maize fewer reads mapped in total, the number of false-positive DGFs varied between species (Supplemental Figure 2A). To overcome this issue, we implemented a more conservative pipeline that, rather than defining false positives at specific locations within the genome, calculates DNaseI cutting bias for all hexamers across each genome. By employing a mixture model framework, these data are then used to generate a background signal to estimate footprint likelihood scores for each putative DGF (Supplemental Figure 2B; Yardımcı et al., 2014). This approach removed between 15 and 30% of DGFs from each sample (Supplemental Figure 2C) and left a total of 430,205 DGFs corresponding to individual transcription factor binding sites between 11 and 25 bp being identified (Figures 1B and 1C; Supplemental Data Set 3). At least one transcription factor footprint was identified in >75% of the broader regions defined by DHSs (Supplemental Data Set 2).

We attempted to saturate the number of predicted DGFs by sequencing each species at high depth (Supplemental Data Set 1). In silico subsampling of these data indicated that for *S. bicolor*, *S. italica*, and *B. distachyon*, the total number of DGFs was close to saturation, but for maize, despite obtaining 251,955,063 reads from whole leaves, this was not the case (Supplemental Figure 3). Consequently, fewer DGFs were predicted in maize compared with the other species (Figure 1C). Since maize has a similar gene number to the other species analyzed, it is possible that the reduced ability to map reads to unique loci was associated with the high amount of repetitive DNA in the maize genome. Another contributing factor to the poor mapping rate in maize may be the low complexity found in one of the libraries, as reflected by the PCR bottleneck coefficient (Supplemental Data Set 1). According to the Encyclopedia of DNA Elements (ENCODE), the large number of reads from low-complexity libraries decreases the chances of identifying the majority of transcription factor binding sites. However, despite these differences in coverage and in certain quality metrics, for all four species, DHSs and DGFs were primarily located in gene-rich regions and depleted around centromeres (Figure 1D). Individual transcription factor binding sequences were resolved in all chromosomes from each species (Figure 1D). On a genome-wide basis, the distribution of DHSs was similar between species, with the highest proportion of such sites located in promoter, coding sequence, and intergenic regions (Figure 1E). Notably, in all four grasses, genic sequences contained more DHSs than promoters (Figure 1F).

**Figure 2.**
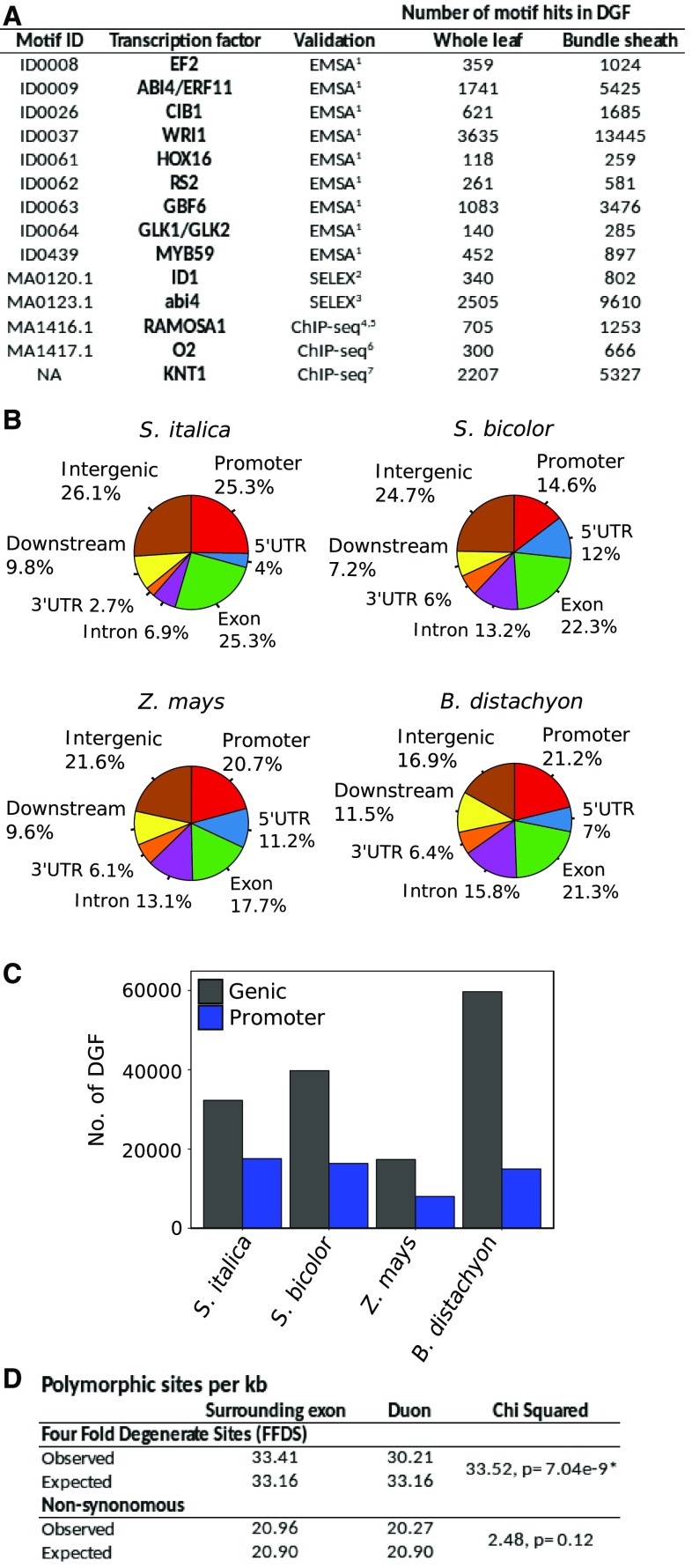
DGFs in Whole Leaves of Four Grasses. **(A)** DNA motifs from previous studies in maize (Niu et al., 2002; Kozaki et al., 2004; Vollbrecht et al., 2005; Bolduc et al., 2012; Eveland et al., 2014; Li et al., 2015; Yu et al., 2015) were detected in whole leaves and bundle-sheath strands from maize. **(B)** Pie chart representing the distribution of DGFs among genomic features. Promoters are defined as sequence up to 2000 bp upstream of the transcriptional start site, downstream represents regions 1000 bp downstream of the transcription termination site, while intergenic represents >1000 bp downstream of the transcription termination site until the next promoter region. UTR, untranslated region. **(C)** Bar charts representing the number of DGFs in genic and promoter regions. **(D)** Polymorphic sites per kb in duons and surrounding exons at FFDS and nonsynonymous sites. χ^2^ tests indicate reduced rates of mutation at FFDS than expected by chance.

To further test whether DGFs identified in our analysis derive from protein-DNA interactions, they were compared with previously identified motifs from maize. Maize is the most appropriate choice for this analysis, as there are more data on transcription factor binding sites than in *S. bicolor* and *S. italica*. Moreover, previous work goes some way to supporting the smaller number of DGFs that we identified in this species. Therefore, the literature was assessed for validated transcription factor binding sites in maize. These have previously been associated with flowering (Kozaki et al., 2004; Vollbrecht et al., 2005; Eveland et al., 2014), meristem development (Bolduc et al., 2012), gibberellin catabolism (Bolduc and Hake, 2009), sugar signaling (Niu et al., 2002), and leaf development of maize (Yu et al., 2015), but in all cases, DGFs matching these motifs were found in our data set (false discovery rate < 0.001; Figure 2A). In addition, a larger chromatin immunoprecipitation (ChIP)-seq data set of 117 transcription factors from maize leaves obtained from the prerelease maize cistrome (http://www.epigenome.cuhk.edu.hk/C3C4.html; Supplemental Figure 4; Supplemental Data Set 4) was compared with our data. Differences between specific binding sites are likely because in all cases growth conditions will have varied from ours, and in some cases different tissues were sampled. Despite this, 66 and 29% of the ChIP-seq peaks overlapped with our DHSs and DGFs, respectively. Although only 29% of DGFs overlapped with motifs defined by ChIP-seq, permutation tests performed using the regioneR package (Gel et al., 2016) indicated a statistically greater overlap than would be expected by chance (P = 0.0099, 100 permutations). Moreover, when both features were systematically shifted from their original position, the local z-score, which represents the strength of the association at any particular position, showed a sharper decrease for DGFs than DHSs, suggesting that the association between ChIP-seq peaks and DGFs is more strongly linked to the exact position of the DGFs (Supplemental Figure 4B). In summary, despite detecting fewer DGFs in maize than in the other species, the DGFs we found are supported by publicly available ChIP-seq, electrophoretic mobility shift assay, and Systematic Evolution of Ligands by EXponential (Selex) data sets.

Consistent with the distribution of DHSs (Figure 1E), annotated DGFs were most common in promoter, coding sequence, and intergenic regions (Figure 2B), and genic sequences contained more DGFs than promoters (Figure 2C). Distribution plots showed that the highest density of DGFs was close to the annotated transcription start sites but indicated a slightly skewed distribution favoring genic sequence including exons (Supplemental Figure 5). A similar pattern was observed for the ChIP-seq signal peaks (Supplemental Figure 4C). Transcription factor binding sites located in exons have been termed duons because they could influence the regulation of both transcription and amino acid sequence. While in general synonymous mutations not affecting amino acid sequence should be under relaxed purifying selection, because of transcription factor recognition, all nucleotides in duons should be under purifying selection and thus show lower mutation rates. We therefore investigated the nucleotide substitution rate at fourfold degenerate sites (FFDS) using variation data from 1218 maize lines (Bukowski et al., 2018) and found that it was statistically significantly lower in duons than in surrounding coding sequence (P = 7.04e-9; Figure 2D). This contrasts with the density of polymorphisms in nonsynonymous sites (Figure 2D). Although it has been proposed that the GC bias of duons constrains FFDS (Xing and He, 2015), we found no such bias between duon and exon sequences used in this analysis (Supplemental Figure 6). Taken together, we conclude that in these cereals a significant proportion of transcription factor binding likely takes place within genes.

### A Distinct *cis*-Regulatory Lexicon for Specific Cells within the Leaf

**Figure 3.**
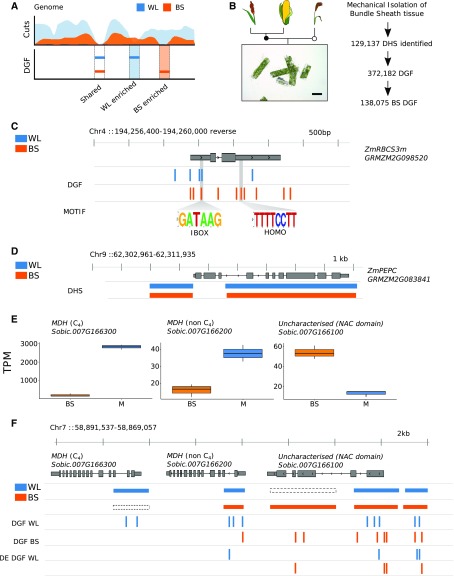
Characterization of the DNA Binding Landscape in the C_4_ Bundle Sheath. **(A)** Schematic showing that due to their negative distribution below the background signal derived from reads mapping to the genome, footprints associated with low-abundance cells such as the bundle sheath (BS) are unlikely to be detected from whole-leaf (WL) samples. **(B)** Bundle-sheath isolation for DNaseI-seq experiments, with phylogeny (left) and workflow (right). **(C)** DGFs identified in the maize *ZmRBCS3* gene coincide with I- and HOMO-boxes known to regulate gene expression. The gene model is annotated with whole-leaf (blue) and bundle-sheath (orange) DGFs, and the I- and HOMO-boxes are indicated below. **(D)** DHS distribution across the maize *PEPC* gene in bundle-sheath and whole-leaf samples. **(E)** Transcript abundance expressed as transcripts per million reads (TPM) of three colinear genes on chromosome 7 of *S. bicolor*, C_4_ *MDH* (Sobic.007G166300), non-C_4_ MDH (non-C_4_; Sobic.007G166200), and an uncharacterized NAC domain protein (Sobic.007G166100), in bundle-sheath and mesophyll cells. Schematics of these colinear genes from *S. bicolor* depict three classes of alterations to DNA accessibility and transcription factor binding to genes that are differentially expressed between whole-leaf and bundle-sheath cells. **(F)** Whole-leaf (blue) and bundle-sheath (orange) DHSs, DGFs, and differentially enriched (DE) DGFs, as determined by the Wellington bootstrap algorithm of the three co-linear genes, are depicted. Regions where a DHS was identified in one sample but not another are indicated by dashed boxes.

The above analysis provides a genome-wide overview of the *cis*-regulatory architecture associated with leaves of grasses. However, as with other complex multicellular systems, leaves are composed of many specialized cell types. Because DGFs are defined by the differential DNA cleavage between protected and unprotected regions of DNA within a DHS, a negative distribution compared with the larger DHS is produced (Figure 3A). Thus, transcription factor binding signal from a low-abundance cell type is likely to be obscured by overall signal from a tissue-level analysis (Figure 3A). Since bundle-sheath strands can be separated (Furbank et al., 1985; Leegood, 1985; Covshoff et al., 2013), C_4_ species provide a simple system to study transcription factor binding in specific cells of leaves (Figure 3B). After bundle-sheath isolation from *S. bicolor*, *S. italica*, and *Z. mays* and naked DNA correction for inherent bias in DNaseI cutting, a total of 129,137 DHSs were identified (Figure 3B; Supplemental Data Set 5) containing 244,554 DGFs (false discovery rate < 0.01; Figure 3B; Supplemental Data Set 5). Of these, 138,075 were statistically enriched in the bundle-sheath samples compared with whole leaves (Figure 3B; Supplemental Data Set 5). The number of these statistically enriched DGFs in bundle-sheath strands of C_4_ species was large and ranged from 14,250 to 73,057 in maize and *S. italica*, respectively (Supplemental Data Set 5). The lower number in maize is likely due to the reduced sequencing depth achieved. Genome-wide, the number of broad regulatory regions defined by DHSs in the bundle sheath that overlapped with those present in whole leaves ranged from 71% to 84% in *S. italica* and *S. bicolor*, respectively (Supplemental Data Set 6). However, only 6% to 20% of the narrower DGFs found in the bundle sheath were also identified in whole leaves (Supplemental Data Set 7). Taken together, these findings indicate that specific cell types of cereal leaves share similarity in the broad regions of DNA that are accessible to transcription factors (DHSs) but that the short sequences actually bound by transcription factors (DGFs) vary dramatically.

To provide evidence that DGFs predicted after analysis of separated bundle-sheath strands are of functional importance, they were compared with previously validated sequences. In C_4_ grasses, we found no such examples in *S. bicolor* or *S. italica*, but in the *RbcS* gene from maize, which is preferentially expressed in bundle-sheath cells, an I-box (GATAAG) is essential for light-mediated activation (Giuliano et al., 1988) and a HOMO motif (CCTTTTTTCCTT) is important in driving bundle-sheath expression (Figure 3C; Xu et al., 2001). Despite not reaching saturation in DGF prediction in maize (Supplemental Figure 3), both elements were detected in our pipeline. Interestingly, a signal suggesting transcription factor binding to the HOMO motif was enriched in the bundle-sheath strands (Figure 3C), and while the I-box was detected in both bundle-sheath strands and whole leaves, its position was slightly different in each cell type (Figure 3C). These findings are therefore consistent with the biochemical data implicating the I-box in the control of abundance and the HOMO-box in the control of cell-specific accumulation of *RbcS* transcripts.

The *ZmPEPC* gene (GRMZM2G083841) encodes the phospho*enol*pyruvate carboxylase responsible for producing C_4_ acids used in the C_4_ pathway and is preferentially expressed in mesophyll cells. Previous reports showed that a region of 600 nucleotides upstream of the transcription start site carrying repeated C-rich sequences was sufficient to drive expression in mesophyll cells of maize (Schäffner and Sheen, 1991; Matsuoka et al., 1994; Taniguchi et al., 2000). Although no DGFs were detected with these C-rich sequences, they are located within a DHS, indicating that they are available for transcription factor binding (Figure 3D). Thus, despite the fact that we had not reached saturation of DGFs in maize, for both *RbcS* and *PEPC*, the regions of DNA accessible to transcription factor binding are consistent with previous reports, and in the case of *RbcS*, DGFs were detected that coincide with known *cis*-elements.

To investigate the relationship between cell-specific gene expression and the positions of DHSs and DGFs, the DNaseI data were interrogated using RNA-seq data sets from mesophyll and bundle-sheath cells of C_4_ leaves (Chang et al., 2012; John et al., 2014; Emms et al., 2016). At least three mechanisms associated with cell-specific gene expression operating around individual genes were identified and can be exemplified using three colinear genes found on chromosome 7 of *S. bicolor*. First, in the *NADP-malate dehydrogenase* (*MDH*) gene, which is highly expressed in mesophyll cells and encodes a protein of the core C_4_ cycle (Figure 3E), a broad DHS site and two DGFs were present in whole leaves but not in bundle-sheath strands (Figure 3F). While the presence of this site indicates accessibility of DNA to transcription factors that could activate expression in mesophyll cells, global analysis of all genes strongly and preferentially expressed in bundle-sheath strands versus whole leaves indicates that presence/absence of a DHS in one cell type is not sufficient to generate cell specificity (Supplemental Figures 7 and 8).

Second, in the next contiguous gene that encodes an additional isoform of MDH also preferentially expressed in mesophyll cells (Figure 3E), a DHS was found in both the whole leaf and bundle-sheath strands, but DGFs within this region differed between cell types (Figure 3F). Thus, despite similarity in DNA accessibility, the binding of particular transcription factors varied between cell types. However, once again, genome-wide analysis indicated that alterations to individual DGFs were not sufficient to explain cell-specific gene expression. For example, only 30% to 40% of all enriched DGFs in the bundle sheath were associated with differentially expressed genes (Supplemental Data Set 8).

Lastly, in the third gene in this region, which encodes a NAC domain transcription factor preferentially expressed in bundle-sheath strands (Figure 3E), differentially enriched DGFs were associated both with regions of the gene that have similar DHSs in each cell type but also a region lacking a DHS in whole leaves compared with bundle-sheath strands (Figure 3E). These three classes of alteration to transcription factor accessibility and binding were detectable in genes encoding core components of the C_4_ cycle in all three species (Supplemental Figures 9 to 11). Overall, we conclude that differences in transcription factor binding between cells of C_4_ leaves are associated with both DNA accessibility defined by broad DHSs as well as fine-scale alterations to transcription factor binding defined by DGFs. Moreover, bundle-sheath strands possessed a distinct regulatory landscape compared with the whole leaf, and in genes encoding enzymes of the C_4_ pathway, multiple transcription factor binding sites differed between bundle-sheath and whole-leaf samples. This finding implies that cell-specific gene expression in C_4_ leaves is mediated by combinatorial effects derived from alterations to gene accessibility as defined by DHSs as well as changes to binding of multiple transcription within these regions.

### DNA Motifs Associated with Cell-Specific Expression

**Figure 4.**
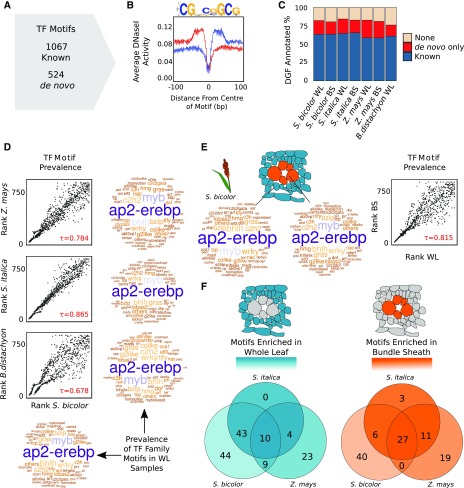
Cistromes Associated with Cell-Specific Gene Expression in C_4_ Grasses. **(A)** Number of previously reported motifs as well as those defined de novo in the grasses. **(B)** Density plots depicting average DNaseI activity on positive (red) and negative (blue) strands centered around a de novo motif. BS, bundle sheath; WL, whole leaf. **(C)** Bar chart depicting percentage of DGFs annotated with known or de novo motifs. **(D)** Comparison of transcription factor (TF) motif prevalence in whole-leaf samples from *S. italica*, *Z. mays*, and *B. distachyon* compared with *S. bicolor*. Word clouds depict the frequency of motifs associated with transcription factor families, with larger names more abundant. Scatterplots compare the frequency of transcription factor motifs within DGFs, ranked from low (most abundant) to high (least abundant). Correlation between samples is indicated as Kendall’s τ coefficient (τ). **(E)** Comparison of transcription factor motif prevalence in bundle-sheath-enriched and whole-leaf-enriched DGFs from *S. bicolor*, as in **(D)**. Word clouds depict the frequency of motifs associated with transcription factor families, and plots compare the frequency of transcription factor motifs within DGFs ranked from low to high. Similarly, scatterplots compare transcription factor motif prevalence in bundle-sheath-enriched and whole-leaf-enriched DGFs from *S. bicolor*. **(F)** Venn diagrams showing enriched motifs for each cell type in all three C_4_ species.

To provide an overview of the transcription factors most likely associated with DGF, ChIP-seq data from maize (Figure 2B) together with motifs from JASPAR plants (Khan et al., 2018) and an additional 529 transcription factor motifs validated in Arabidopsis (*Arabidopsis thaliana*; O’Malley et al., 2016) were used to annotate the DGFs from *Z. mays*, *S. bicolor*, *S. italica*, and *B. distachyon* (Figure 4A). To increase the number of annotated DGFs, de novo prediction was used to identify sequences overrepresented in DGFs compared with those across the whole genome. This resulted in an additional 524 motifs being annotated (Figure 4A), but in fact, all of these were previously detected after de novo prediction from DNaseI-seq of rice (Zhang et al., 2012b). As would be expected from bona fide transcription factor binding, inspection of these motifs predicted de novo demonstrated clear strand bias in DNaseI cuts (Figure 4B). By combining previously known motifs and those predicted de novo, the percentage of DGFs that could be annotated in each species increased from ∼60% to more than 75% (Figure 4C; Supplemental Data Set 9).

To define the most common sequences bound by transcription factors in mature leaves undertaking C_3_ and C_4_ photosynthesis and to investigate whether C_4_ photosynthesis is controlled by an increase in binding of sets of transcription factors, individual motifs were ranked by frequency and the Kendall rank correlation coefficient used to compare species (Figure 4D). In both C_3_ and C_4_ species, the most prevalent transcription factor binding motifs were associated with the AP2-EREBP and MYB transcription factor families (P < 2.2^−16^; Figure 4D). Next, to identify regulatory factors associated with gene expression in the C_4_ bundle sheath, transcription factor motifs located in DGFs enriched in either the bundle sheath or in whole-leaf samples of *S. bicolor* were identified (Figure 4E). There was little difference in the ranking of the most commonly used motifs between these cell types (Kendall’s τ = 0.815; P < 2.2^−16^), indicating that cell specificity is not associated with large-scale changes in the abundance of many transcription factor families (Figure 4E). After performing hypergeometric tests for enrichment of individual motifs in differentially occupied DGFs, we found 133 and 106 motifs enriched in whole leaves and bundle-sheath strands, respectively (P < 0.001). Of these 239 motifs, 37 were enriched in all C_4_ species, with 10 and 27 enriched in the bundle sheath and whole leaf, respectively (Figure 4F; Supplemental Data Set 10); 66 were only enriched in bundle-sheath strands and 91 in whole-leaf tissue (Supplemental Data Set 11). Some of these conserved and cell-specific motifs have been previously described to have a relevant role in photosynthesis. For instance, in whole leaves of maize and *S. italica*, we found significant enrichment of the bHLH129 motif (Supplemental Data Set 11) that has been proposed to act as a negative regulator of NADP-ME (Borba et al., 2018).

### Multiple Genes Encoding Enzymes of the C_4_ and Calvin-Benson-Bassham Cycles Share the Same Occupied *cis*-Elements

To investigate whether genes involved in the C_4_ phenotype are coregulated, we compared the number of instances where the same motifs were bound in multiple C_4_, Calvin-Benson-Bassham, and C_2_ cycle genes (Supplemental Data Set 12). While no single *cis*-element was found in all genes that are preferentially expressed in mesophyll or bundle-sheath cells, the number of genes possessing the same occupied motif ranged from nine in *S. bicolor* and *S. italica* to four in *S. bicolor* and *Z. mays* whole leaves (Supplemental Data Sets 9 and 12). These data support a model where the combinatorial action of multiple transcription factors controls groups of C_4_ genes to produce the gene expression patterns required for C_4_ photosynthesis.

**Figure 5.**
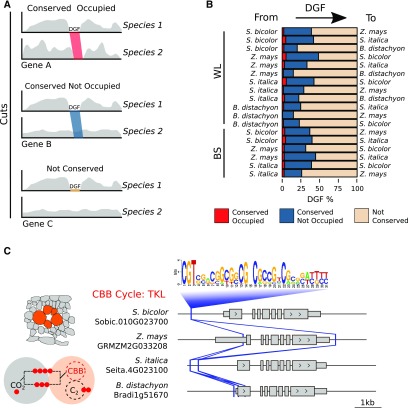
*Cis*-Elements Show High Rates of Turnover and Mobility in Grasses. **(A)** Scenarios for DGF conservation between species. Reads derived from DNaseI cuts are depicted in gray, DGFs that are both conserved and occupied between species are depicted in red, and DGFs that are conserved but unoccupied are depicted in blue shading. **(B)** Bar plot representing pairwise comparisons of DGF occupancy. BS, bundle sheath; WL, whole leaf. **(C)** Schematic depicting the positions of a transcription factor motif consistently associated with the bundle-sheath-enriched *TKL* gene in *S. bicolor*, *Z. mays*, *S. italica*, and C_3_ *B. distachyon*. The positions of motifs conserved between orthologous genes are depicted by solid lines and vary between species.

We next performed comparative analysis of motifs bound by transcription factors to determine whether the set of *cis*-elements found in C_4_ genes of each species were common or whether C_4_ genes are regulated differently in each species. In pairwise comparisons, DGFs fell into three categories: conserved and occupied by a transcription factor, conserved but only occupied in one species, and not conserved (Figure 5A). Only a small percentage of DGFs were both conserved in sequence and bound by transcription factors (Figure 5B; Supplemental Data Set 13). Consistent with this, the majority of C_4_ gene orthologs did not share DGFs. Due to the lack of DGF saturation in maize, these estimates likely set lower bounds for the extent of conservation. However, in several cases, patterning of C_4_ gene expression correlated with a set of motifs shared across species (Figure 5C). In some cases, these shared *cis*-elements were present in the ancestral C_3_ state. For instance, the *TRANSKETOLASE* (*TKL*) gene contains several conserved DGFs that are present in the bundle sheath of the C_4_ species but also in whole leaves of C_3_ *B. distachyon* (Figure 5). This finding is consistent with the notion that C_4_ photosynthesis makes use of existing regulatory architecture found in C_3_ plants. Nevertheless, overall, these data also indicate that the majority of C_4_ gene expression appears to be associated with species-specific regulatory networks.

### Hyperconserved *cis*-Regulators of C_4_ Genes

**Figure 6.**
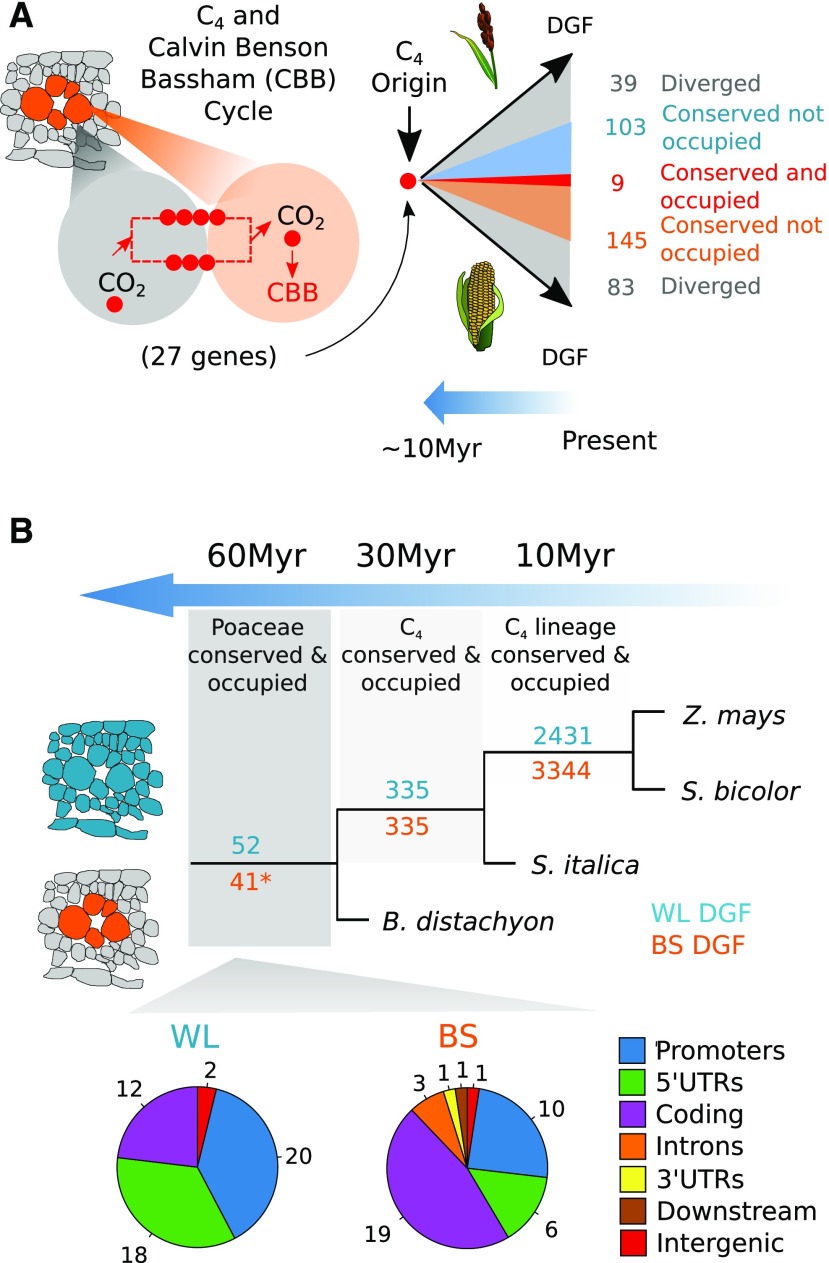
Hyperconserved *cis*-Elements in Grasses Recruited into C_4_ Photosynthesis. **(A)** Conservation of regulation in C_4_ and Calvin-Benson-Bassham cycle genes following the divergence of *Z. mays* and *S. bicolor*. The number of carbon atoms (red dots) and metabolite flow (red dashed line) between mesophyll (gray) and bundle-sheath (orange) cells are illustrated along with the degree of conservation of DGFs associated with bundle-sheath strands. **(B)** Conservation of DGF occupancy in grasses across evolutionary time. Results are depicted for whole-leaf (WL; blue) and bundle-sheath (BS; orange) DGFs. The asterisk indicates 41 DGFs that are conserved in the bundle sheath of the C_4_ species but are also found in whole leaves of *B. distachyon*. Pie charts display the distribution of conserved and occupied DGFs for the whole-leaf and bundle-sheath strands. Promoters are defined as sequence up to 2000 bp upstream of the transcriptional start site, downstream represents regions 1000 bp downstream of the transcription termination site, while intergenic represents >1000 bp downstream of the transcription termination site until the next promoter region. Myr, million years; UTR, untranslated region.

To investigate the extent to which transcription factor binding sites associated with C_4_ genes within a C_4_ lineage are conserved, genes encoding the core C_4_ cycle were compared in *S. bicolor* and *Z. mays* (Figure 6A; Supplemental Data Set 14). Twenty-seven genes associated with the C_4_ and Calvin-Benson-Bassham cycles contained a total of 379 DGFs. Although many of these transcription factor footprints were conserved in sequence within orthologous genes, only nine were both conserved and bound by a transcription factor (Figure 6A). Again, due to the lack of DGF saturation in maize, these data likely represent minimum estimates of conservation.

Genome-wide, the number of DGFs that were conserved in sequence and bound by a transcription factor decayed in a nonlinear manner with phylogenetic distance (Figure 6B; Supplemental Data Set 15). For example, *Z. mays* and *S. bicolor* shared 5775 DGFs that were both conserved and occupied. *S. italica* shared only 670 DGFs with *Z. mays* and *S. bicolor* (Figure 6B). Finally, comparison of these C_4_ grasses with C_3_ *B. distachyon* yielded 93 DGFs that have been conserved over >60 million years of evolution. Because nuclei from *B. distachyon* were sampled later in the photoperiod than those from the C_4_ grasses and DGFs may well vary over the diel cycle, it is possible that this is an underestimate of DGF conservation. However, 41 of these highly conserved DGFs were present in whole-leaf samples of the C_3_ species, but in the C_4_ species they were restricted to the bundle sheath (Figure 6B). Gene Ontology analysis did not detect enrichment of any specific terms for hyperconserved DGFs associated with the bundle sheath, but for whole leaves, it detected overrepresentation of “cell component” categories such as membrane-bound organelles and the nucleus (Supplemental Data Set 16). In whole leaves, this set of ancient and highly conserved DGFs were located predominantly in 5ʹ untranslated regions and coding sequences, but in bundle-sheath strands, over 50% of these hyperconserved DGFs were in coding sequences (Figure 6B). Overall, these data indicate that certain duons are highly conserved across deep evolutionary time. The frequent use of hyperconserved duons in the bundle sheath implies that this cell type uses an ancient and highly conserved regulatory code.

## DISCUSSION

### Genome-Wide Transcription Factor Binding in Grasses

The data set provides insight into the regulation of gene expression in cereals in general and to C_4_ photosynthesis in particular. Consistent with previous analyses ranging from Arabidopsis (Sullivan et al., 2014) to metazoans (Natarajan et al., 2012; Stergachis et al., 2013, 2014), the majority of DGFs detected in the four grasses were centered around annotated transcription start sites. However, in these cereals, it is noteworthy that transcription factor binding was prevalent in genic sequences. While we cannot rule out the possibility that this distribution is in some way related to the methodology used in this study, there is evidence that the exact distribution of transcription factor binding appears to be species specific. For example, while in Arabidopsis DNaseI-seq revealed enrichment of DHSs in sequence ∼400 bp upstream of transcription start sites as well as 5ʹ untranslated regions (Sullivan et al., 2014) and assay for transposase-accessible chromatin of Arabidopsis, *Medicago truncatula*, and rice detected most transposase-hypersensitive sites upstream of genes, in *Solanum lycopersicum*, more were present in introns and exons than upstream of annotated transcription start sites (Maher et al., 2018).

The prevalence of transcription factor binding to coding sequences is relevant to approaches used to generate transgenic plants and test gene function and regulation. First, consistent with the prevalence of DGFs downstream of the annotated transcription start sites that we detected, it is noteworthy that during cereal transformation, exon and intron sequences are frequently used to achieve stable expression of transgenes (Maas et al., 1991; Cornejo et al., 1993; Jeon et al., 2000). It is possible that this strategy is required in grasses because of the high proportion of transcription factor binding downstream of annotated transcription start sites. These transcription factor binding sites in coding sequence also have implications for synthetic biology. Although technologies such as type IIS restriction endonuclease cloning methods allow high-throughput testing of many transgenes, they rely on sequence domestication. While routinely this would maintain amino acid sequence, without analysis of transcription factor binding sites it could mutate motifs bound by transcription factors and lead to unintended modifications to gene expression.

### The Transcription Factor Landscape Underpinning Gene Expression in Specific Tissues

The finding that so few transcription factor binding sites were shared between bundle-sheath tissue and whole leaves of *S. bicolor*, *Z. mays*, and *S. italica* argues for the need to isolate these cells when attempting to understand the control of gene expression. Although separating bundle-sheath strands from C_4_ leaves is relatively trivial (Furbank et al., 1985; Leegood, 1985; Covshoff et al., 2013), this is not the case for C_3_ leaves. Approaches in which nuclei from specific cell types are labeled with an exogenous tag (Deal and Henikoff, 2011) now allow their transcription factor landscapes to be defined. The application of chromatin accessibility assays to specific cell types has recently been used in roots (Maher et al., 2018), and so in the future, this approach of both C_3_ and C_4_ leaves should provide insight into the extent to which gene regulatory networks have been rewired during the evolution of the complex C_4_ trait.

Given the central importance of cellular compartmentation to C_4_ photosynthesis, there have been significant efforts to identify *cis*-elements that restrict gene expression to either mesophyll or bundle-sheath cells of C_4_ leaves (Sheen, 1999; Hibberd and Covshoff, 2010; Wang et al., 2014). As previous studies of C_4_ gene regulation have focused on individual genes and have been performed in various species, it has not been possible to obtain a coherent picture of regulation of the C_4_ pathway, and along with many other systems, initial analyses focused on regulatory elements located in promoters of C_4_ genes (Sheen, 1999). However, it has become increasingly apparent that the patterning of gene expression between cells in the C_4_ leaf can be mediated by elements in various parts of a gene. In addition to promoter elements (Sheen, 1999; Gowik et al., 2004), this includes untranslated regions (Viret et al., 1994; Xu et al., 2001; Patel et al., 2004; Kajala et al., 2012; Williams et al., 2016) and coding sequences (Brown et al., 2011; Reyna-Llorens et al., 2018). By providing data on in vivo transcription factor occupancy for the complete C_4_ pathway in three C_4_ grasses, the data presented here allow broad comparisons and provide several insights into regulatory networks controlling C_4_ genes.

The DNaseI data set indicates that cell-specific gene expression in C_4_ leaves is not strongly correlated with changes to large-scale accessibility of DNA as defined by DHSs. This implies that modifications to transcription factor accessibility around any one gene do not influence its expression between tissues in the leaf. Rather, as only 8 to 24% of transcription factor binding sites detected in the bundle sheath were also found in whole leaves, the data strongly implicate complex modifications to patterns of transcription factor binding in controlling gene expression between cell types. These findings are consistent with analogous analyses in roots where genes with clear spatial patterns of expression are bound by multiple transcription factors (Sparks et al., 2016) and highly combinatorial interactions between multiple activators and repressors tune the output (de Lucas et al., 2016).

The data also provide insight into *cis*-elements that underpin the C_4_ phenotype. No single c*is*-element was found in all genes preferentially expressed in either mesophyll or bundle-sheath cells of one species. This finding is consistent with analyses of yeast, where the output of genetic circuits can be maintained despite rapid turnover of *cis-*regulatory mechanisms underpinning them (Tsong et al., 2006). However, we did detect small numbers of C_4_ genes that shared common transcription factor footprints (Figure 5; Supplemental Data Sets 14 and 15), which is consistent with previous analyses that identified shared *cis*-elements in *PPDK* and *CA* or *NAD-ME1* and *NAD-ME2* in C_4_ *Gynandropsis gynandra* (Williams et al., 2016; Reyna-Llorens et al., 2018). Interspecific comparisons further underlined the high rate of divergence in the *cis*-regulatory logic used to control C_4_ genes. For example, although we detected highly similar transcription factor footprints in the *OMT1* and *TKL* genes of the three C_4_ species we assessed, this was not apparent for any other C_4_ genes. As a result of the apparent rapid rate of evolution in *cis*-regulatory architecture in these C_4_ species, attempts to engineer C_4_ photosynthesis into C_3_ crops to increase yield (Hibberd et al., 2008) may benefit from using preexisting regulatory mechanisms controlling mesophyll or bundle-sheath expression in ancestral C_3_ species.

### Characteristics of the Transcription Factor Binding in the Ancestral C_3_ State That Have Influenced the Evolution of the C_4_ Pathway

Comparison of transcription factor binding in the C_3_ grass *B. distachyon* with three C_4_ species provides insight into mechanisms associated with the evolution of C_4_ photosynthesis. For all four grasses, irrespective of whether they used C_3_ or C_4_ photosynthesis, the most abundant DNA motifs bound by transcription factors were similar. Thus, motifs recognized by the AP2-EREBP and MYB classes of transcription factor were most commonly bound across each genome. This indicates that during the evolution of C_4_ photosynthesis, there has been relatively little alteration to the most abundant classes of transcription factors that bind DNA.

The repeated evolution of the C_4_ pathway has frequently been associated with convergent evolution (Sage, 2004; Sage et al., 2012). However, parallel alterations to amino acid and nucleotide sequences that allow altered kinetics of the C_4_ enzymes (Christin et al., 2007; Christin and Osborne, 2014) and patterning of C_4_ gene expression (Brown et al., 2011), respectively, have also been reported. The genome-wide analysis of transcription factor binding reported here indicates that only a small proportion of the C_4_ cistrome is associated with parallel evolution. These estimates regarding conservation between C_4_ and C_3_ species may represent underestimates, because while nuclei were all sampled in the light, those from *B. distachyon* were sampled later in the photoperiod. Moreover, when orthologous genes were compared between the four grasses assessed here, the majority of transcription factor binding sites were not conserved, and of the DGFs that were conserved, position within orthologous genes varied. This indicates that C_4_ photosynthesis in grasses is tolerant to a rapid turnover of the *cis*-code and that when motifs are conserved in sequence, their position and frequency within a gene can vary. It therefore appears that the cell-specific accumulation patterns of C_4_ proteins can be maintained despite considerable modifications to the cistrome of C_4_ leaves. It was also the case that some conserved motifs bound by transcription factors in the C_4_ species were present in *B. distachyon*, which uses the ancestral C_3_ pathway. Previous work has shown that *cis*-elements used in C_4_ photosynthesis can be found in gene orthologs from C_3_ species (Williams et al., 2016; Reyna-Llorens et al., 2018). However, these previous studies identified *cis*-elements that were conserved in both sequence and position. As it is now clear that such conserved motifs are mobile within a gene, it seems likely that many more examples of ancient *cis*-elements important in C_4_ photosynthesis will be found in C_3_ plants.

Although we were able to detect a small number of transcription factor binding sites that were conserved and occupied in all four species sampled, these ancient hyperconserved motifs appear to have played a role in the evolution of C_4_ photosynthesis. Interestingly, a large proportion of these motifs bound by transcription factors were found in coding sequences, and this bias was particularly noticeable in bundle-sheath cells. Due to the amino acid code, the rate of mutation of coding sequence compared with the genome is restricted. If such regions have a longer half-life than transcription factor binding sites in other regions of the genome, then they may represent an excellent source of raw material for the repeated evolution of complex traits (Martin and Orgogozo, 2013). Our data documenting the frequent use of hyperconserved DGFs in the C_4_ bundle sheath imply that this tissue may use an ancient and highly conserved regulatory code. It appears that during the evolution of the C_4_ pathway, which relies on heavy use of the bundle sheath, this ancient code has been coopted to control photosynthesis gene expression.

In summary, the data provide a transcription factor binding atlas for leaves of grasses using either C_3_ or C_4_ photosynthesis. While we did not achieve DGF saturation in maize, commonalities between the four species were apparent. Sequences bound by transcription factors were found within genes as well as promoter regions, and many of these motifs represent duons. In terms of the regulation of tissue-specific gene expression, while bundle-sheath strands and whole C_4_ leaves shared considerable similarity in regions of DNA accessible to transcription factors, the short sequences actually bound by transcription factors varied dramatically. We identified a small number of transcription factor motifs that were conserved in these species. The data also provide insight into the regulatory architecture associated with C_4_ photosynthesis more specifically. While we found some evidence that multiple genes important for C_4_ photosynthesis share common *cis*-elements bound by transcription factors, this was not widespread. This may well relate to the relatively rapid turnover in the *cis*-code, and so it is possible that transcription factors interacting with these motifs are more conserved. Analysis of transcription factor footprints in specific cell types from leaves of C_3_ grasses should in the future provide insight into the extent to which gene regulatory networks have altered during the transition from C_3_ to C_4_ photosynthesis.

## METHODS

### Growth Conditions and Isolation of Nuclei

*Sorghum bicolor*, *Setaria italica*, and *Zea mays* were grown under controlled conditions at the Plant Growth Facilities of the Department of Plant Sciences at the University of Cambridge in a chamber set to 12 h/12 h light/dark, 28°C light/20°C dark, 400 µmol m^−2^ s^−1^ photon flux density from metal halide 400-W light bulbs, and 60% humidity. For germination, *S. bicolor* and *Z. mays* seeds were imbibed in water for 48 h, and *S. italica* seeds were incubated on wet filter paper at 30°C overnight in the dark. *Z. mays*, *S. bicolor*, and *S. italica* were grown on 3:1 (v/v) M3 compost:medium vermiculite mixture, with a thin covering of soil. Seedlings were hand watered.

*Brachypodium distachyon* plants were grown in a separate growth facility under controlled conditions optimized for its growth at the Sainsbury Laboratory at Cambridge University, first under short-day conditions (14 h/10 h light/dark for 2 weeks) and then shifted to long-day conditions (20 h/4 h light/dark for 1 week), and harvested at Zeitgeber time 20. Temperature was set at 20°C, humidity at 65%, and light intensity at 350 µmol m^−2^ s^−1^. All tissue was harvested from August to October 2015.

To isolate nuclei from *S. bicolor*, *Z. mays*, and *S. italica*, mature third and fourth leaves with a fully developed ligule were harvested 4 to 6 h into the light cycle 18 d after germination. Bundle-sheath cells were mechanically isolated as described previously by Markelz et al. (2003). At least 3 g of tissue was used for each extraction. Nuclei were isolated using a sucrose gradient adapted and yield quantified using a hemocytometer. For *B. distachyon*, plants were flash-frozen and material was pulverized in a coffee grinder. Three grams of plant material was added to 45 mL of nuclei isolation buffer (10 mM Tris-HCl, 0.2 M Suc, and 0.01% [v/v] Triton X-100, pH 5.3, containing protease inhibitors [Sigma-Aldrich]) and incubated at 4°C on a rotating wheel for 5 min, after which debris was removed by sieving through two layers of Miracloth (Millipore) into precooled flasks. Nuclei were spun down at 2862*g* at 4°C for 20 min. Plastids were lysed by adding Triton X-100 to a final concentration of 0.3% (v/v) and incubated for 15 min on ice. Nuclei were pelleted by centrifugation at 4500*g* at 4°C for 15 min. Pellets were washed three times with chilled nuclei isolation buffer.

### Deproteinized DNA Extraction

For isolation of deproteinated DNA from *S. bicolor*, *Z. mays*, *B. distachyon*, and *S. italica*, mature third and fourth leaves with a fully developed ligule were harvested 4 h into the light cycle 18 d after germination. A total of 100 mg of tissue was used for each extraction. Deproteinated DNA was extracted using a DNeasy Plant Mini Kit (Qiagen) according to the manufacturer’s instructions.

### DNaseI Digestion, Sequencing, and Library Preparation

To obtain sufficient DNA, each biological replicate consisted of leaves from tens of individuals, and to conform to standards set by the Human Genome Project, at least two biological replicates were sequenced for each sample. A total of 2 × 10^8^ freshly extracted nuclei were resuspended at 4°C in digestion buffer (15 mM Tris-HCl, 90 mM NaCl, 60 mM KCl, 6 mM CaCl_2_, 0.5 mM spermidine, 1 mM EDTA, and 0.5 mM EGTA, pH 8.0). DNaseI (Fermentas) at 7.5 units was added to each tube and incubated at 37°C for 3 min. Digestion was arrested with the addition of a 1:1 volume of stop buffer (50 mM Tris-HCl, 100 mM NaCl, 0.1% [w/v] SDS, 100 mM EDTA, pH 8.0, 1 mM spermidine, 0.3 mM spermine, and 40 µg/mL RNase A) and incubated at 55°C for 15 min. Fifty units of proteinase K was added, and samples were incubated at 55°C for 1 h. DNA was isolated with 25:24:1 phenol:chloroform:isoamyl alcohol (Ambion) followed by ethanol precipitation. Fragments from 50 to 550 bp were selected using agarose gel electrophoresis. The extracted DNA samples were quantified fluorometrically with a Qubit 3.0 Fluorometer (Life Technologies), and a total of 10 ng of digested DNA (200 pg/L) was used for library construction.

Initial sample quality control of prefragmented DNA was assessed using a Tapestation DNA 1000 High Sensitivity Screen Tape (Agilent). Sequencing-ready libraries were prepared using the Hyper Prep DNA Library preparation kit (Kapa Biosystems) selecting fragments from 70 to 350 bp for optimization (He et al., 2014) and indexed for pooling using NextFlex DNA barcoded adapters (Bioo Scientific). To reduce bias due to amplification of DNA fragments by the PCR, as recommended by the manufacturers, a low number of cycles (17 cycles) was used. Libraries were quantified using a Tapestation DNA 1000 Screen Tape and by qPCR using an Next Generation Sequencing Library Quantification Kit (KAPA Biosystems) on an AriaMx qPCR system (Agilent) and then normalized, pooled, diluted, and denatured for sequencing on the NextSeq 500 (Illumina). The main library was spiked at 10% with the PhiX control library (Illumina). Sequencing was performed using Illumina NextSeq in the Departments of Biochemistry and Pathology at the University of Cambridge with 2× 75 cycles of sequencing. For the deproteinized DNaseI-seq experiments, 1 µg of deproteinized DNA was resuspended in 1 mL of digestion buffer (15 mM Tris-HCl, 90 mM NaCl, 60 mM KCl, 6 mM CaCl_2_, 0.5 mM spermidine, 1 mM EDTA, and 0.5 mM EGTA, pH 8.0). DNaseI (Fermentas) at 2.5 units was added to each tube and incubated at 37°C for 2 min. Digestion was arrested with the addition of a 1:1 volume of stop buffer (50 mM Tris-HCl, 100 mM NaCl, 0.1% [w/v] SDS, 100 mM EDTA, pH 8.0, 1 mM spermidine, 0.3 mM spermine, and 40 µg/mL RNase A) and incubated at 55°C for 15 min. Fifty units of proteinase K was added, and samples were incubated at 55°C for 1 h. DNA was isolated by mixing with 1 mL of 25:24:1 phenol:chloroform:isoamyl alcohol (Ambion) and spun for 5 min at 15,700*g* followed by ethanol precipitation of the aqueous phase. Samples were then size-selected (50–400 bp) using agarose gel electrophoresis. The extracted DNA samples were quantified fluorometrically using a Qubit 3.0 Fluorometer (Life Technologies), and a total of 1 ng of digested DNA was used for library construction. Sequencing-ready libraries were prepared using a KAPA Hyper Prep Kit (KAPA Biosystems) according to the manufacturer’s instructions. To reduce bias due to amplification of DNA fragments by the PCR, as recommended by the manufacturers, 17 cycles were used. The quality of the libraries was checked using a Bioanalyzer High Sensitivity DNA Chip (Agilent Technologies). Libraries were quantified with a Qubit 3.0 Fluorometer (Life Technologies) and qPCR using an Next Generation Sequencing Library Quantification Kit (KAPA Biosystems) and then normalized, pooled, diluted, and denatured for paired end sequencing using high-output 150-cycle run (2× 75-bp reads). Sequencing was performed using NextSeq 500 (Illumina) in the Sainsbury Laboratory at the University of Cambridge with 2× 75 cycles of sequencing.

### DNaseI-Seq Data Processing

Genome sequences were downloaded from Phytozome (v10; Goodstein et al., 2012). The following genome assemblies were used: Bdistachyon_283_assembly_v2.0, Sbicolor_255_v2.0, Sitalica_164_v2, and Zmays_284_AGPv3. Due to the lack of guidelines for DNaseI-seq experiments in plants, we followed the guidelines from ENCODE3. Reads were mapped to genomes using bowtie2 (Langmead and Salzberg, 2012) and processed using samtools (Li et al., 2009) to remove those with a MAPQ score < 42. DHSs were called using MACS2 (Feng et al., 2012), and the final set of peak calls were determined using the irreproducible discovery rate (Li and Dewey, 2011), calculated using the script batch_consistency_analysis.R (https://github.com/modENCODE-DCC/Galaxy/blob/master/modENCODE_DCC_tools/idr/batch-consistency-analysis.r). The irreproducible discovery rate framework adapted from the ENCODE3 pipeline (Marinov et al., 2014; https://sites.google.com/site/anshulkundaje/projects/idr) aims to measure the reproducibility of findings by identifying the point (threshold) at which peaks are no longer consistent across replicates.

### Quality Metrics and Identification of DGFs

The SPOT score (number of a subsample of mapped reads [5M] in DHSs/total number of subsampled, mapped reads [5M]; John et al., 2011) was calculated using BEDTools (Quinlan and Hall, 2010) to determine the number of mapped reads possessing at least 1 bp of overlap with a DHS site. Normalized strand cross-correlation coefficient and relative strand cross-correlation coefficient scores were calculated using SPP (Kharchenko et al., 2008), and PCR bottleneck coefficient was calculated using BEDTools. To account for cutting bias associated with the DNaseI enzyme, DNaseI-seq on naked DNA was performed. These data were used to generate background signal profiles and calculate the footprint log-likelihood ratio for each footprint using the R package MixtureModel (Yardımcı et al., 2014), such that those with low log-likelihood ratios (<0) were removed. DGFs were identified using Wellington (Piper et al., 2013), and differential DGFs were identified using Wellington bootstrap (Piper et al., 2015).

### Data Visualization

DHS and DGF sequences were loaded into and visualized in the Integrative Genomics Viewer (Thorvaldsdóttir et al., 2013) and figures produced in Inkscape; plots were generated with the R package ggplot2 (Wickham, 2010) and figures depicting conservation of DGFs or motifs between orthologous sequences were generated using genoplotR (Guy et al., 2010). Word clouds were created with the wordcloud R package (Fellows, 2012). TreeView images were produced by processing DGF data using dnase_to_javatreeview.py from pyDNase (Piper et al., 2013, 2015) and loaded into TreeView (Saldanha, 2004). Average cut density plots were generated using the script dnase_average_profile.py from pyDNase. Genomic features were annotated and distribution calculated using PAVIS (Huang et al., 2013) and plotted using ggplot2. For each gene, promoter regions were defined as sequence 2000 bp upstream of the transcriptional start site, and downstream regions were defined as 1000 bp subsequent to the transcription termination site. C_4_, C_2_, and Calvin-Benson-Bassham cycle gene orthologs were selected on the basis of transcript abundance from previous studies (Chang et al., 2012; John et al., 2014; Emms et al., 2016). Circular plots showing the distribution of ChIP-seq peaks, DHSs, and DGFs across the maize genome were generated using the R package circlize (Gu et al., 2014).

### DNase Cutting Bias Calculations and ChIP-Seq Analysis

After sequencing, the number of DNA 6-mers centered at each DNase cleavage site (between third and fourth bases) was counted and normalized by the total number of counts. Next, DNA 6-mer frequencies were normalized by the frequencies of each DNA 6-mer in the genome. The resulting background signal profile was used as input in the FootprintMixture.R package (https://ohlerlab.mdc-berlin.de/software/FootprintMixture_109/; Supplemental Figure 2).

ChIP-seq peaks from 117 transcription factors were obtained from the prerelease maize cistrome data collection (http://www.epigenome.cuhk.edu.hk/C3C4.html). Permutation tests between ChIP peaks and DHSs or DGFs were performed using regioneR (Gel et al., 2016) using 100 permutations.

### De Novo Motif Prediction, Motif Scanning, and Enrichment Testing

De novo motif prediction was performed using findMotifsGenome.pl script from the HOMER suite (Heinz et al., 2010) using DGFs as input together with the reference genome sequence for each species. Motif scanning was performed using FIMO (Grant et al., 2011) with default parameters. To determine overrepresentation of transcription factor family motifs in samples, hypergeometric tests were performed using R. The distribution of each motif across different genomic features was obtained for each annotated motif by dividing the number of hits in a particular feature by the total number of hits in the genome.

### Whole-Genome Alignments, Pairwise Cross-Mapping of Genomic Features, and Variant Data Processing

To cross-map genomic features between species, mapping files were generated according to http://genomewiki.ucsc.edu/index.php/Whole_genome_alignment_howto using tools from the University of California, Santa Cruz Genome Browser, including trfBig, faToNib, faSize, lavToPsl, faSplit, axtChain, chainNet (Kent et al., 2002), and LASTZ (Harris, 2007). Briefly, whole-genome alignment was performed with LASTZ; matching alignments next to each other were chained together using axtChain, sorted with axtSort, and then netted together to form larger blocks with chainNet. Genomic features were then mapped between genomes using bnMapper (Denas et al., 2015). For the variant analysis on duons, *Z. mays* variant data (Bukowski et al., 2018) was downloaded from https://sites.google.com/site/anshulkundaje/projects/idr/deprecated following instructions. After downloading, vcf files were annotated using SnpEff (Cingolani et al., 2012; https://doi.org/10.4161/fly.19695) with the B73_RefGen_v4 genome assembly specifically to allow identification of nonsynonymous sites. A custom script was used to identify all FFDS in the *Z. mays* genome. This bed file in turn was used to identify which of the synonymous polymorphic sites were FFDS. Each polymorphic site had its allele frequencies calculated. Putative *Z. mays* duons were identified by intersecting (with BEDTools intersect) the final DGFs identified with exonic regions. These duons were then used to extract only those exons within which a duon was found. These exons in turn had the duon regions themselves subtracted to leave the exon region except the duon. This provided the surrounding exonic sequences with which to compare the duons. These two regions were then intersected with the polymorphism data to identify both the number of occurrences and allelic frequencies of polymorphic sites (FFDS and nonsynonymous) within both the duons and their surrounding exonic sequences.

### Accession Numbers

Methods for DNaseI digestion are on protocols.io (10.17504/protocols.io.hdfb23n). Raw sequencing data and processed files are deposited in the Gene Expression Omnibus (GSE97369) and The National Center for Biotechnology Information (PRJNA381532). For full methods, commands, and scripts, as well as processed data to be loaded into a genome browser, see github (https://github.com/hibberd-lab/Burgess-Reyna_llorens-monocot-DNase) and Figshare 10.6084/m9.figshare.7649450.

### Supplemental Data

**Supplemental Figure 1.** DNaseI digestion of nuclei for sequencing.
**Supplemental Figure 2.** Bias in DNaseI-SEQ cleavage.
**Supplemental Figure 3.** Saturation analysis of footprints.
**Supplemental Figure 4.** Genome-wide comparison of DGF and ChIP-SEQ peaks from 117 maize transcription factors.
**Supplemental Figure 5.** Density plot depicting the distribution of DGFs per kilobase (kb) from the transcription start site (TSS) of *S. bicolor*, *Z. mays*, *S. italica* and *B. distachyon* whole leaves.
**Supplemental Figure 6.** Nucleotide proportion of duons and surrounding exons used in the substitution analysis for *Z. mays*.
**Supplemental Figure 7.** Transcript abundance for genes in mesophyll and bundle-sheath cells associated with DHSs and DGFs in *S. bicolor*.
**Supplemental Figure 8.** Differential accessibility of broad regulatory regions in *S. bicolor* is not sufficient for cell preferential gene expression.
**Supplemental Figure 9.** Representation of the C_4_ pathway showing differentially accessible DHSs, DGFs and cell-specific DGFs between whole-leaf (blue) and bundle-sheath (orange) samples in *S. bicolor*.
**Supplemental Figure 10.** Representation of the C_4_ pathway showing differentially accessible DHSs, DGFs and cell-specific DGFs between whole-leaf (blue) and bundle-sheath (orange) samples in *S. italica*.
**Supplemental Figure 11.** Representation of the C_4_ pathway showing differentially accessible DHSs, DGFs and cell-specific DGFs between whole-leaf (blue) and bundle-sheath (orange) samples in *Z. mays*.
**Supplemental Data Set 1.** Summary of DNaseI-SEQ quality metrics.
**Supplemental Data Set 2.** Summary statistics for genomic features identified in whole-leaf samples.
**Supplemental Data Set 3.** DNaseI cutting bias calculation summary for whole-leaf and bundle-sheath data.
**Supplemental Data Set 4.** Transcription factors included in the ChIP-SEQ data analysis.
**Supplemental Data Set 5.** Summary statistics of DNaseI-SEQ analysis of bundle-sheath samples
**Supplemental Data Set 6.** Summary statistics of overlap between DHSs in whole-leaf and bundle-sheath samples.
**Supplemental Data Set 7.** Summary statistics of overlap between DGFs in whole-leaf and bundle-sheath samples.
**Supplemental Data Set 8.** Summary statistics for differential digital genomic footprint calling.
**Supplemental Data Set 9.** Motifs mapped to genes of the C_4_, CBB and C_2_ cycles in *Z. mays*, *S. bicolor*, *S. italica* for whole-leaf and bundle-sheath samples and in *B. distachyon* for whole-leaf samples.
**Supplemental Data Set 10.** Hypergeometric tests for enrichment of individual motifs in *Z. mays*, *S. bicolor*, *S. italica* for whole-leaf and bundle-sheath samples.
**Supplemental Data Set 11.** Hypergeometric tests for enrichment of cell-specific individual motifs in *Z. mays*, *S. bicolor*, *S. italica* for whole-leaf and bundle-sheath samples.
**Supplemental Data Set 12.** Number of genes in C_4_, CBB and C_2_ cycles annotated with a given motif in *Z. mays*, *S. italica*, *S. bicolor* and *B. distachyon*.
**Supplemental Data Set 12.** Statistics for cross mapping of genomic features between *S. bicolor*, *S. italica*, *Z. mays* and *B. distachyon*.
**Supplemental Data Set 13.** DGFs conserved and occupied in *Z. mays*, *S. bicolor*, *S. italica* for whole-leaf and bundle-sheath samples and in *B. distachyon* for whole-leaf samples.
**Supplemental Data Set 14.** DGFs in C_4_ genes that are conserved between *Z. mays* and *S. bicolor*.
**Supplemental Data Set 15.** DGFs conserved in all four species.
**Supplemental Data Set 16.** Gene Ontology analysis on hyper-conserved DGFs in whole-leaf samples of *S. italica*, *S. bicolor*, *Z. mays* and *B. distachyon*.

## Dive Curated Terms

The following phenotypic, genotypic, and functional terms are of significance to the work described in this paper:

DHS Gramene: LOC_Os02g45780
DHS Araport: LOC_Os02g45780

SDS Gramene: AT1G14750
SDS Araport: AT1G14750

dna Gramene: EO:0007532
dna Araport: EO:0007532

## References

[bib1] Bauwe, H., Hagemann, M., Fernie, A.R. (2010). Photorespiration: Players, partners and origin. Trends Plant Sci. 15: 330–336. 20403720 10.1016/j.tplants.2010.03.006

[bib2] Bolduc, N., Hake, S. (2009). The maize transcription factor KNOTTED1 directly regulates the gibberellin catabolism gene *ga2ox1*. Plant Cell 21: 1647–1658. 19567707 10.1105/tpc.109.068221 PMC2714931

[bib3] Bolduc, N., Yilmaz, A., Mejia-Guerra, M.K., Morohashi, K., O’Connor, D., Grotewold, E., Hake, S. (2012). Unraveling the KNOTTED1 regulatory network in maize meristems. Genes Dev. 26: 1685–1690. 22855831 10.1101/gad.193433.112 PMC3418586

[bib4] Borba, A.R., Serra, T.S., Górska, A., Gouveia, P., Cordeiro, A.M., Reyna-Llorens, I., Knerová, J., Barros, P.M., Abreu, I.A., Oliveira, M.M., Hibberd, J.M., Saibo, N.J.M. (2018). Synergistic binding of bHLH transcription factors to the promoter of the maize NADP-ME gene used in C4 photosynthesis is based on an ancient code found in the ancestral C3 state. Mol. Biol. Evol. 35: 1690–1705. 29659975 10.1093/molbev/msy060 PMC5995220

[bib5] Bowes, G., Ogren, W.L., Hageman, R.H. (1971). Phosphoglycolate production catalyzed by ribulose diphosphate carboxylase. Biochem. Biophys. Res. Commun. 45: 716–722. 4331471 10.1016/0006-291x(71)90475-x

[bib6] Brown, N.J., Newell, C.A., Stanley, S., Chen, J.E., Perrin, A.J., Kajala, K., Hibberd, J.M. (2011). Independent and parallel recruitment of preexisting mechanisms underlying C_4_ photosynthesis. Science 331: 1436–1439. 21415351 10.1126/science.1201248

[bib7] Bukowski, R., . (2018). Construction of the third-generation *Zea mays* haplotype map. Gigascience 7: 1–12. 10.1093/gigascience/gix134 PMC5890452 29300887

[bib8] Chang, Y.M., Liu, W.Y., Shih, A.C.C., Shen, M.N., Lu, C.H., Lu, M.Y.J., Yang, H.W., Wang, T.Y., Chen, S.C., Chen, S.M., Li, W.H., Ku, M.S. (2012). Characterizing regulatory and functional differentiation between maize mesophyll and bundle sheath cells by transcriptomic analysis. Plant Physiol. 160: 165–177. 22829318 10.1104/pp.112.203810 PMC3440195

[bib9] Christin, P.A., Osborne, C.P. (2014). The evolutionary ecology of C_4_ plants. New Phytol. 204: 765–781. 25263843 10.1111/nph.13033

[bib10] Christin, P.A., Salamin, N., Savolainen, V., Duvall, M.R., Besnard, G. (2007). C_4_ photosynthesis evolved in grasses via parallel adaptive genetic changes. Curr. Biol. 17: 1241–1247. 17614282 10.1016/j.cub.2007.06.036

[bib11] Cingolani, P., Platts, A., Wang, L., Coon, M., Nguyen, T., Wang, L., Land, S.J., Lu, X., Ruden, D.M. (2012). A program for annotating and predicting the effects of single nucleotide polymorphisms, SnpEff: SNPs in the genome of *Drosophila melanogaster* strain w1118; iso-2; iso-3. Fly (Austin) 6: 80–92. 22728672 10.4161/fly.19695 PMC3679285

[bib12] Cornejo, M.-J., Luth, D., Blankenship, K.M., Anderson, O.D., Blechl, A.E. (1993). Activity of a maize ubiquitin promoter in transgenic rice. Plant Mol. Biol. 23: 567–581. 8219091 10.1007/BF00019304

[bib13] Covshoff, S., Furbank, R.T., Leegood, R.C., Hibberd, J.M. (2013). Leaf rolling allows quantification of mRNA abundance in mesophyll cells of sorghum. J. Exp. Bot. 64: 807–813. 23077203 10.1093/jxb/ers286

[bib14] Deal, R.B., Henikoff, S. (2011). The INTACT method for cell type-specific gene expression and chromatin profiling in *Arabidopsis thaliana*. Nat. Protoc. 6: 56–68. 21212783 10.1038/nprot.2010.175 PMC7219316

[bib15] de Lucas, M., Pu, L., Turco, G., Gaudinier, A., Morao, A.K., Harashima, H., Kim, D., Ron, M., Sugimoto, K., Roudier, F., Brady, S.M. (2016). Transcriptional regulation of Arabidopsis Polycomb Repressive Complex 2 coordinates cell-type proliferation and differentiation. Plant Cell 28: 2616–2631. 27650334 10.1105/tpc.15.00744 PMC5134969

[bib16] Denas, O., Sandstrom, R., Cheng, Y., Beal, K., Herrero, J., Hardison, R.C., Taylor, J. (2015). Genome-wide comparative analysis reveals human-mouse regulatory landscape and evolution. BMC Genomics 16: 87. 25765714 10.1186/s12864-015-1245-6 PMC4333152

[bib17] Emms, D.M., Covshoff, S., Hibberd, J.M., Kelly, S. (2016). Independent and parallel evolution of new genes by gene duplication in two origins of C4 photosynthesis provides new insight into the mechanism of phloem loading in C4 species. Mol. Biol. Evol. 33: 1796–1806. 27016024 10.1093/molbev/msw057 PMC4915358

[bib18] Eveland, A.L., . (2014). Regulatory modules controlling maize inflorescence architecture. Genome Res. 24: 431–443. 24307553 10.1101/gr.166397.113 PMC3941108

[bib19] Fellows, I. (2012). wordcloud: Word clouds. R Package; version 2: 109. https://cran.r-project.org/web/packages/wordcloud/wordcloud.pdf.

[bib20] Feng, J., Liu, T., Qin, B., Zhang, Y., Liu, X.S. (2012). Identifying ChIP-seq enrichment using MACS. Nat. Protoc. 7: 1728–1740. 22936215 10.1038/nprot.2012.101 PMC3868217

[bib21] Furbank, R.T. (2011). Evolution of the C_4_ photosynthetic mechanism: Are there really three C_4_ acid decarboxylation types? J. Exp. Bot. 62: 3103–3108. 21511901 10.1093/jxb/err080

[bib22] Furbank, R.T., Stitt, M., Foyer, C.H. (1985). Intercellular compartmentation of sucrose synthesis in leaves of *Zea mays* L. Planta 164: 172–178. 24249558 10.1007/BF00396079

[bib23] Gel, B., Díez-Villanueva, A., Serra, E., Buschbeck, M., Peinado, M.A., Malinverni, R. (2016). regioneR: An R/Bioconductor package for the association analysis of genomic regions based on permutation tests. Bioinformatics 32: 289–291. 26424858 10.1093/bioinformatics/btv562 PMC4708104

[bib24] Giuliano, G., Pichersky, E., Malik, V.S., Timko, M.P., Scolnik, P.A., Cashmore, A.R. (1988). An evolutionarily conserved protein binding sequence upstream of a plant light-regulated gene. Proc. Natl. Acad. Sci. USA 85: 7089–7093. 2902624 10.1073/pnas.85.19.7089 PMC282129

[bib25] Goodstein, D.M., Shu, S., Howson, R., Neupane, R., Hayes, R.D., Fazo, J., Mitros, T., Dirks, W., Hellsten, U., Putnam, N., Rokhsar, D.S. (2012). Phytozome: A comparative platform for green plant genomics. Nucleic Acids Res. 40: D1178–D1186. 22110026 10.1093/nar/gkr944 PMC3245001

[bib26] Gowik, U., Burscheidt, J., Akyildiz, M., Schlue, U., Koczor, M., Streubel, M., Westhoff, P. (2004). *cis*-Regulatory elements for mesophyll-specific gene expression in the C_4_ plant *Flaveria trinervia*, the promoter of the C_4_ *phosphoenolpyruvate carboxylase* gene. Plant Cell 16: 1077–1090. 15100398 10.1105/tpc.019729 PMC423201

[bib27] Grant, C.E., Bailey, T.L., Noble, W.S. (2011). FIMO: Scanning for occurrences of a given motif. Bioinformatics 27: 1017–1018. 21330290 10.1093/bioinformatics/btr064 PMC3065696

[bib28] Gu, Z., Gu, L., Eils, R., Schlesner, M., Brors, B. (2014). circlize implements and enhances circular visualization in R. Bioinformatics 30: 2811–2812. 24930139 10.1093/bioinformatics/btu393

[bib29] Guy, L., Kultima, J.R., Andersson, S.G.E. (2010). genoPlotR: Comparative gene and genome visualization in R. Bioinformatics 26: 2334–2335. 20624783 10.1093/bioinformatics/btq413 PMC2935412

[bib30] Harris, R.S. (2007). Improved Pairwise Alignment of Genomic DNA. PhD dissertation (State College, PA: Pennsylvania State University).

[bib31] Hatch, M.D. (1987). C_4_ photosynthesis: A unique blend of modified biochemistry, anatomy and ultrastructure. Biochim. Biophys. Acta Rev. Bioenerg. 895: 81–106.

[bib32] He, H.H., Meyer, C.A., Hu, S.S., Chen, M.W., Zang, C., Liu, Y., Rao, P.K., Fei, T., Xu, H., Long, H., Liu, X.S., Brown, M. (2014). Refined DNase-seq protocol and data analysis reveals intrinsic bias in transcription factor footprint identification. Nat. Methods 11: 73–78. 24317252 10.1038/nmeth.2762 PMC4018771

[bib33] Heinz, S., Benner, C., Spann, N., Bertolino, E., Lin, Y.C., Laslo, P., Cheng, J.X., Murre, C., Singh, H., Glass, C.K. (2010). Simple combinations of lineage-determining transcription factors prime cis-regulatory elements required for macrophage and B cell identities. Mol. Cell 38: 576–589. 20513432 10.1016/j.molcel.2010.05.004 PMC2898526

[bib34] Hesselberth, J.R., Chen, X., Zhang, Z., Sabo, P.J., Sandstrom, R., Reynolds, A.P., Thurman, R.E., Neph, S., Kuehn, M.S., Noble, W.S., Fields, S., Stamatoyannopoulos, J.A. (2009). Global mapping of protein-DNA interactions in vivo by digital genomic footprinting. Nat. Methods 6: 283–289. 19305407 10.1038/nmeth.1313 PMC2668528

[bib35] Hibberd, J.M., Covshoff, S. (2010). The regulation of gene expression required for C_4_ photosynthesis. Annu. Rev. Plant Biol. 61: 181–207. 20192753 10.1146/annurev-arplant-042809-112238

[bib36] Hibberd, J.M., Sheehy, J.E., Langdale, J.A. (2008). Using C4 photosynthesis to increase the yield of rice-rationale and feasibility. Curr. Opin. Plant Biol. 11: 228–231. 18203653 10.1016/j.pbi.2007.11.002

[bib37] Huang, W., Loganantharaj, R., Schroeder, B., Fargo, D., Li, L. (2013). PAVIS: A tool for Peak Annotation and Visualization. Bioinformatics 29: 3097–3099. 24008416 10.1093/bioinformatics/btt520 PMC3834791

[bib38] Jeon, J.-S., Lee, S., Jung, K.-H., Jun, S.-H., Kim, C., An, G. (2000). Tissue-preferential expression of a rice α-tubulin gene, *OsTubA1*, mediated by the first intron. Plant Physiol. 123: 1005–1014. 10889249 10.1104/pp.123.3.1005 PMC59063

[bib39] John, C.R., Smith-Unna, R.D., Woodfield, H., Covshoff, S., Hibberd, J.M. (2014). Evolutionary convergence of cell-specific gene expression in independent lineages of C4 grasses. Plant Physiol. 165: 62–75. 24676859 10.1104/pp.114.238667 PMC4012605

[bib40] John, S., Sabo, P.J., Thurman, R.E., Sung, M.-H., Biddie, S.C., Johnson, T.A., Hager, G.L., Stamatoyannopoulos, J.A. (2011). Chromatin accessibility pre-determines glucocorticoid receptor binding patterns. Nat. Genet. 43: 264–268. 21258342 10.1038/ng.759 PMC6386452

[bib41] Jordan, D.B., Ogren, W.L. (1984). The CO_2_/O_2_ specificity of ribulose 1,5-bisphosphate carboxylase/oxygenase: Dependence on ribulosebisphosphate concentration, pH and temperature. Planta 161: 308–313. 24253719 10.1007/BF00398720

[bib42] Kajala, K., Brown, N.J., Williams, B.P., Borrill, P., Taylor, L.E., Hibberd, J.M. (2012). Multiple Arabidopsis genes primed for recruitment into C_4_ photosynthesis. Plant J. 69: 47–56. 21883556 10.1111/j.1365-313X.2011.04769.x

[bib43] Kent, W.J., Sugnet, C.W., Furey, T.S., Roskin, K.M., Pringle, T.H., Zahler, A.M., Haussler, D. (2002). The human genome browser at UCSC. Genome Res. 12: 996–1006. 12045153 10.1101/gr.229102 PMC186604

[bib44] Khan, A., . (2018). JASPAR 2018: Update of the open-access database of transcription factor binding profiles and its web framework. Nucleic Acids Res. 46: D260–D266. 29140473 10.1093/nar/gkx1126 PMC5753243

[bib45] Kharchenko, P.V., Tolstorukov, M.Y., Park, P.J. (2008). Design and analysis of ChIP-seq experiments for DNA-binding proteins. Nat. Biotechnol. 26: 1351–1359. 19029915 10.1038/nbt.1508 PMC2597701

[bib46] Kozaki, A., Hake, S., Colasanti, J. (2004). The maize ID1 flowering time regulator is a zinc finger protein with novel DNA binding properties. Nucleic Acids Res. 32: 1710–1720. 15020707 10.1093/nar/gkh337 PMC390334

[bib47] Langmead, B., Salzberg, S.L. (2012). Fast gapped-read alignment with Bowtie 2. Nat. Methods 9: 357–359. 22388286 10.1038/nmeth.1923 PMC3322381

[bib48] Leegood, R.C. (1985). The intercellular compartmentation of metabolites in leaves of *Zea mays* L. Planta 164: 163–171. 24249557 10.1007/BF00396078

[bib49] Li, B., Dewey, C.N. (2011). RSEM: Accurate transcript quantification from RNA-Seq data with or without a reference genome. BMC Bioinformatics 12: 323. 21816040 10.1186/1471-2105-12-323 PMC3163565

[bib50] Li, C., Qiao, Z., Qi, W., Wang, Q., Yuan, Y., Yang, X., Tang, Y., Mei, B., Lv, Y., Zhao, H., Xiao, H., Song, R. (2015). Genome-wide characterization of cis-acting DNA targets reveals the transcriptional regulatory framework of opaque2 in maize. Plant Cell 27: 532–545. 25691733 10.1105/tpc.114.134858 PMC4558662

[bib51] Li, H., Handsaker, B., Wysoker, A., Fennell, T., Ruan, J., Homer, N., Marth, G., Abecasis, G., Durbin, R.; 1000 Genome Project Data Processing Subgroup (2009). The Sequence Alignment/Map format and SAMtools. Bioinformatics 25: 2078–2079. 19505943 10.1093/bioinformatics/btp352 PMC2723002

[bib52] Maas, C., Laufs, J., Grant, S., Korfhage, C., Werr, W. (1991). The combination of a novel stimulatory element in the first exon of the maize *Shrunken-1* gene with the following intron 1 enhances reporter gene expression up to 1000-fold. Plant Mol. Biol. 16: 199–207. 1893097 10.1007/BF00020552

[bib53] Maher, K.A., . (2018). Profiling of accessible chromatin regions across multiple plant species and cell types reveals common gene regulatory principles and new control modules. Plant Cell 30: 15–36. 29229750 10.1105/tpc.17.00581 PMC5810565

[bib54] Marinov, G.K., Kundaje, A., Park, P.J., Wold, B.J. (2014). Large-scale quality analysis of published ChIP-seq data. G3 (Bethesda) 4: 209–223. 24347632 10.1534/g3.113.008680 PMC3931556

[bib55] Markelz, N.H., Costich, D.E., Brutnell, T.P. (2003). Photomorphogenic responses in maize seedling development. Plant Physiol. 133: 1578–1591. 14645729 10.1104/pp.103.029694 PMC300715

[bib56] Martin, A., Orgogozo, V. (2013). The loci of repeated evolution: A catalog of genetic hotspots of phenotypic variation. Evolution 67: 1235–1250. 23617905 10.1111/evo.12081

[bib57] Matsuoka, M., Kyozuka, J., Shimamoto, K., Kano-Murakami, Y. (1994). The promoters of two carboxylases in a C4 plant (maize) direct cell-specific, light-regulated expression in a C3 plant (rice). Plant J. 6: 311–319. 7920719 10.1046/j.1365-313x.1994.06030311.x

[bib58] Natarajan, A., Yardimci, G.G., Sheffield, N.C., Crawford, G.E., Ohler, U. (2012). Predicting cell-type-specific gene expression from regions of open chromatin. Genome Res. 22: 1711–1722. 22955983 10.1101/gr.135129.111 PMC3431488

[bib59] Neph, S., . (2012). An expansive human regulatory lexicon encoded in transcription factor footprints. Nature 489: 83–90. 22955618 10.1038/nature11212 PMC3736582

[bib60] Niu, X., Helentjaris, T., Bate, N.J. (2002). Maize ABI4 binds coupling element1 in abscisic acid and sugar response genes. Plant Cell 14: 2565–2575. 12368505 10.1105/tpc.003400 PMC151236

[bib61] O’Malley, R.C., Huang, S.C., Song, L., Lewsey, M.G., Bartlett, A., Nery, J.R., Galli, M., Gallavotti, A., Ecker, J.R. (2016). Cistrome and epicistrome features shape the regulatory DNA landscape. Cell 165: 1280–1292. 27203113 10.1016/j.cell.2016.04.038 PMC4907330

[bib62] Pajoro, A., . (2014). Dynamics of chromatin accessibility and gene regulation by MADS-domain transcription factors in flower development. Genome Biol. 15: R41. 24581456 10.1186/gb-2014-15-3-r41 PMC4054849

[bib63] Patel, M., Corey, A.C., Yin, L.P., Ali, S., Taylor, W.C., Berry, J.O. (2004). Untranslated regions from C_4_ amaranth *AhRbcS1* mRNAs confer translational enhancement and preferential bundle sheath cell expression in transgenic C_4_ *Flaveria bidentis*. Plant Physiol. 136: 3550–3561. 15489276 10.1104/pp.104.051508 PMC527154

[bib64] Pautler, M., Eveland, A.L., LaRue, T., Yang, F., Weeks, R., Lunde, C., Je, B.I., Meeley, R., Komatsu, M., Vollbrecht, E., Sakai, H., Jackson, D. (2015). *FASCIATED EAR4* encodes a bZIP transcription factor that regulates shoot meristem size in maize. Plant Cell 27: 104–120. 25616871 10.1105/tpc.114.132506 PMC4330574

[bib65] Piper, J., Elze, M.C., Cauchy, P., Cockerill, P.N., Bonifer, C., Ott, S. (2013). Wellington: A novel method for the accurate identification of digital genomic footprints from DNase-seq data. Nucleic Acids Res. 41: e201. 24071585 10.1093/nar/gkt850 PMC3834841

[bib66] Piper, J., Assi, S.A., Cauchy, P., Ladroue, C., Cockerill, P.N., Bonifer, C., Ott, S. (2015). Wellington-bootstrap: Differential DNase-seq footprinting identifies cell-type determining transcription factors. BMC Genomics 16: 1000. 26608661 10.1186/s12864-015-2081-4 PMC4658755

[bib67] Quinlan, A.R., Hall, I.M. (2010). BEDTools: A flexible suite of utilities for comparing genomic features. Bioinformatics 26: 841–842. 20110278 10.1093/bioinformatics/btq033 PMC2832824

[bib68] Reyna-Llorens, I., Burgess, S.J., Reeves, G., Singh, P., Stevenson, S.R., Williams, B.P., Stanley, S., Hibberd, J.M. (2018). Ancient duons may underpin spatial patterning of gene expression in C_4_ leaves. Proc. Natl. Acad. Sci. USA 115: 1931–1936. 29432183 10.1073/pnas.1720576115 PMC5828626

[bib69] Sage, R. (2004). The evolution of C_4_ photosynthesis. New Phytol. 161: 341–370. 33873498 10.1111/j.1469-8137.2004.00974.x

[bib70] Sage, R.F., Zhu, X.G. (2011). Exploiting the engine of C_4_ photosynthesis. J. Exp. Bot. 62: 2989–3000. 21652533 10.1093/jxb/err179

[bib71] Sage, R.F., Christin, P.-A., Edwards, E.J. (2011). The C_4_ plant lineages of planet Earth. J. Exp. Bot. 62: 3171–3181. 21414957 10.1093/jxb/err048

[bib72] Sage, R.F., Sage, T.L., Kocacinar, F. (2012). Photorespiration and the evolution of C_4_ photosynthesis. Annu. Rev. Plant Biol. 63: 19–47. 22404472 10.1146/annurev-arplant-042811-105511

[bib73] Saldanha, A.J. (2004). Java Treeview: Extensible visualization of microarray data. Bioinformatics 20: 3246–3248. 15180930 10.1093/bioinformatics/bth349

[bib74] Schäffner, A.R., Sheen, J. (1991). Maize rbcS promoter activity depends on sequence elements not found in dicot rbcS promoters. Plant Cell 3: 997–1012. 1822995 10.1105/tpc.3.9.997 PMC160066

[bib75] Sharwood, R.E., Ghannoum, O., Kapralov, M.V., Gunn, L.H., Whitney, S.M. (2016). Temperature responses of Rubisco from Paniceae grasses provide opportunities for improving C_3_ photosynthesis. Nat. Plants 2: 16186. 27892943 10.1038/nplants.2016.186

[bib76] Sheen, J. (1999). C_4_ gene expression. Annu. Rev. Plant Physiol. Plant Mol. Biol. 50: 187–217. 15012208 10.1146/annurev.arplant.50.1.187

[bib77] Sparks, E.E., . (2016). Establishment of expression in the SHORTROOT-SCARECROW transcriptional cascade through opposing activities of both activators and repressors. Dev. Cell 39: 585–596. 27923776 10.1016/j.devcel.2016.09.031 PMC5349323

[bib78] Stergachis, A.B., . (2014). Conservation of trans-acting circuitry during mammalian regulatory evolution. Nature 515: 365–370. 25409825 10.1038/nature13972 PMC4405208

[bib79] Stergachis, A.B., Haugen, E., Shafer, A., Fu, W., Vernot, B., Reynolds, A., Raubitschek, A., Ziegler, S., LeProust, E.M., Akey, J.M., Stamatoyannopoulos, J.A. (2013). Exonic transcription factor binding directs codon choice and affects protein evolution. Science 342: 1367–1372. 24337295 10.1126/science.1243490 PMC3967546

[bib80] Sullivan, A.M., . (2014). Mapping and dynamics of regulatory DNA and transcription factor networks in *A. thaliana*. Cell Rep. 8: 2015–2030. 25220462 10.1016/j.celrep.2014.08.019

[bib81] Taniguchi, M., Izawa, K., Ku, M.S.B., Lin, J.H., Saito, H., Ishida, Y., Ohta, S., Komari, T., Matsuoka, M., Sugiyama, T. (2000). Binding of cell type-specific nuclear proteins to the 5′-flanking region of maize C_4_ phosphoenolpyruvate carboxylase gene confers its differential transcription in mesophyll cells. Plant Mol. Biol. 44: 543–557. 11197328 10.1023/a:1026565027772

[bib82] Thorvaldsdóttir, H., Robinson, J.T., Mesirov, J.P. (2013). Integrative Genomics Viewer (IGV): High-performance genomics data visualization and exploration. Brief. Bioinform. 14: 178–192. 22517427 10.1093/bib/bbs017 PMC3603213

[bib83] Thurman, R.E., . (2012). The accessible chromatin landscape of the human genome. Nature 489: 75–82. 22955617 10.1038/nature11232 PMC3721348

[bib84] Tolbert, N.E. (1971). Microbodies: Peroxisomes and glyoxysomes. Annu. Rev. Plant Physiol. 22: 45–74.

[bib85] Tsong, A.E., Tuch, B.B., Li, H., Johnson, A.D. (2006). Evolution of alternative transcriptional circuits with identical logic. Nature 443: 415–420. 17006507 10.1038/nature05099

[bib86] Viret, J.F., Mabrouk, Y., Bogorad, L. (1994). Transcriptional photoregulation of cell-type-preferred expression of maize *rbcS-m3*: 3′ and 5′ sequences are involved. Proc. Natl. Acad. Sci. USA 91: 8577–8581. 8078926 10.1073/pnas.91.18.8577 PMC44649

[bib87] Vollbrecht, E., Springer, P.S., Goh, L., Buckler, E.S., IV, Martienssen, R. (2005). Architecture of floral branch systems in maize and related grasses. Nature 436: 1119–1126. 16041362 10.1038/nature03892

[bib88] Wang, L., Czedik-Eysenberg, A., Mertz, R.A., Si, Y., Tohge, T., Nunes-Nesi, A., Arrivault, S., Dedow, L.K., Bryant, D.W., Zhou, W., Xu, J., Weissmann, S., (2014). Comparative analyses of C₄ and C₃ photosynthesis in developing leaves of maize and rice. Nat. Biotechnol. 32: 1158–1165. 25306245 10.1038/nbt.3019

[bib89] Wickham, H. (2010). ggplot2: Elegant graphics for data analysis. J. Stat. Softw. 35: 65–68.

[bib90] Williams, B.P., Burgess, S.J., Reyna-Llorens, I., Knerova, J., Aubry, S., Stanley, S., Hibberd, J.M. (2016). An untranslated *cis*-element regulates the accumulation of multiple C_4_ enzymes in *Gynandropsis gynandra* mesophyll cells. Plant Cell 28: 454–465. 26772995 10.1105/tpc.15.00570 PMC4790868

[bib91] Xing, K., He, X. (2015). Reassessing the “duon” hypothesis of protein evolution. Mol. Biol. Evol. 32: 1056–1062. 25582593 10.1093/molbev/msu409

[bib92] Xu, T., Purcell, M., Zucchi, P., Helentjaris, T., Bogorad, L. (2001). TRM1, a YY1-like suppressor of *rbcS-m3* expression in maize mesophyll cells. Proc. Natl. Acad. Sci. USA 98: 2295–2300. 11226233 10.1073/pnas.041610098 PMC30132

[bib93] Yardımcı, G.G., Frank, C.L., Crawford, G.E., Ohler, U. (2014). Explicit DNase sequence bias modeling enables high-resolution transcription factor footprint detection. Nucleic Acids Res. 42: 11865–11878. 25294828 10.1093/nar/gku810 PMC4231734

[bib94] Yu, C.P., . (2015). Transcriptome dynamics of developing maize leaves and genomewide prediction of *cis* elements and their cognate transcription factors. Proc. Natl. Acad. Sci. USA 112: E2477–E2486. 25918418 10.1073/pnas.1500605112 PMC4434728

[bib95] Zentner, G.E., Henikoff, S. (2014). High-resolution digital profiling of the epigenome. Nat. Rev. Genet. 15: 814–827. 25297728 10.1038/nrg3798

[bib96] Zhang, T., Marand, A.P., Jiang, J. (2016). PlantDHS: A database for DNase I hypersensitive sites in plants. Nucleic Acids Res. 44: D1148–D1153. 26400163 10.1093/nar/gkv962 PMC4702941

[bib97] Zhang, W., Wu, Y., Schnable, J.C., Zeng, Z., Freeling, M., Crawford, G.E., Jiang, J. (2012 a). High-resolution mapping of open chromatin in the rice genome. Genome Res. 22: 151–162. 22110044 10.1101/gr.131342.111 PMC3246202

[bib98] Zhang, W., Zhang, T., Wu, Y., Jiang, J. (2012 b). Genome-wide identification of regulatory DNA elements and protein-binding footprints using signatures of open chromatin in Arabidopsis. Plant Cell 24: 2719–2731. 22773751 10.1105/tpc.112.098061 PMC3426110

